# A novel procedure for transtracheal resection for recurrent thyroid cancer involving a trachea and esophagus

**DOI:** 10.1186/1477-7819-12-303

**Published:** 2014-10-02

**Authors:** Shinichi Ohba, Junkichi Yokoyama, Mitsuhisa Fujimaki, Masataka Kojima, Katsuhisa Ikeda

**Affiliations:** Department of Otorhinolaryngology-Head and Neck Surgery, Juntendo University School of Medicine, 2-1-1, Hongo, Bunkyo-ku, Tokyo, 113-8421 Japan

**Keywords:** function preservation, minimally invasive surgery, poorly differentiated thyroid carcinoma, recurrent thyroid cancer, transtracheal surgery

## Abstract

**Background:**

Surgery remains the main treatment for locally advanced thyroid cancers invading the trachea, esophagus, and recurrent laryngeal nerve. However, extensive resection of such tumors can sometimes involve difficulties and may result in the deterioration of the patient’s quality of life. The surgeon should consider not only the patient’s prognosis but also the preservation of postoperative function.

**Methods:**

This report describes a minimally invasive surgical procedure for recurrent poorly differentiated papillary thyroid carcinoma involving the trachea and esophagus. To decrease the potential for recurrent laryngeal nerve injuries and to preserve both the tracheal and esophageal blood supply, we adapted a transtracheal approach; the recurrent tumor was safely and completely removed without causing a dysfunction. After a tracheotomy to the right, the tumor was easily detected through the tracheostoma and delineated by palpation. The mucous membrane of the trachea was minimally incised along the right-hand border of the tumor and a mucosal flap was elevated. The left side of the trachea including the membranous wall and cartilage of the tracheal mucosa was maximally preserved, to maintain the vascular supply to the trachea. Finally, the membranous wall of the trachea was preserved to within one-third of the left-hand side. Furthermore, the risk of bleeding from major lateral vessels was reduced. A sternocleidomastoid muscle flap was elevated and inserted into the cavity resulting from the tumor resection and sutured between the esophagus and trachea. The membranous wall of the tracheal mucosa was also sutured submucosally.

**Results:**

The tumor was removed completely with the muscular layer of the esophagus without injury to the intact recurrent laryngeal nerve and lateral major vessels. The patient started oral nutritional intake on the first postoperative day and was discharged without any significant postoperative complications.

**Conclusions:**

This new procedure for transtracheal resection for recurrent thyroid cancer involving the trachea and esophagus was useful and safe.

## Background

Surgery remains the mainstay for the treatment of locally advanced recurrent thyroid cancer. However, with complex recurrent thyroid cancer with recurrent laryngeal nerve palsy, dissection for the trachea and esophagus can be associated with significant morbidity, including dyspnea and dysphagia [1, 2]. The morbidity from radical resection has led some surgeons to avoid curative treatments. Our objective was to perform minimally invasive surgery with transtracheal central neck dissection for recurrent thyroid cancer. The key point of this technique was to complete the procedure with potentially less risk of injury to the intact recurrent laryngeal nerve or the lateral major vessels. To our knowledge, there have been no reports regarding minimally invasive surgery for the treatment of recurrent thyroid cancer.

## Methods

### Case presentation

**Figure 1:**
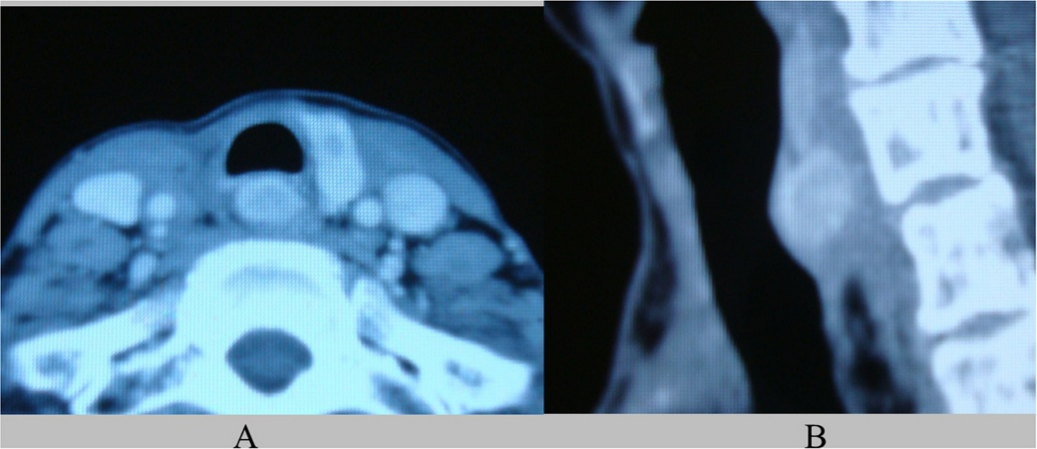
**CT of neck with contrast medium, showing heterogeneous enhanced mass between trachea and esophagus. (A)** axial section; **(B)** sagittal section.

**Figure 2:**
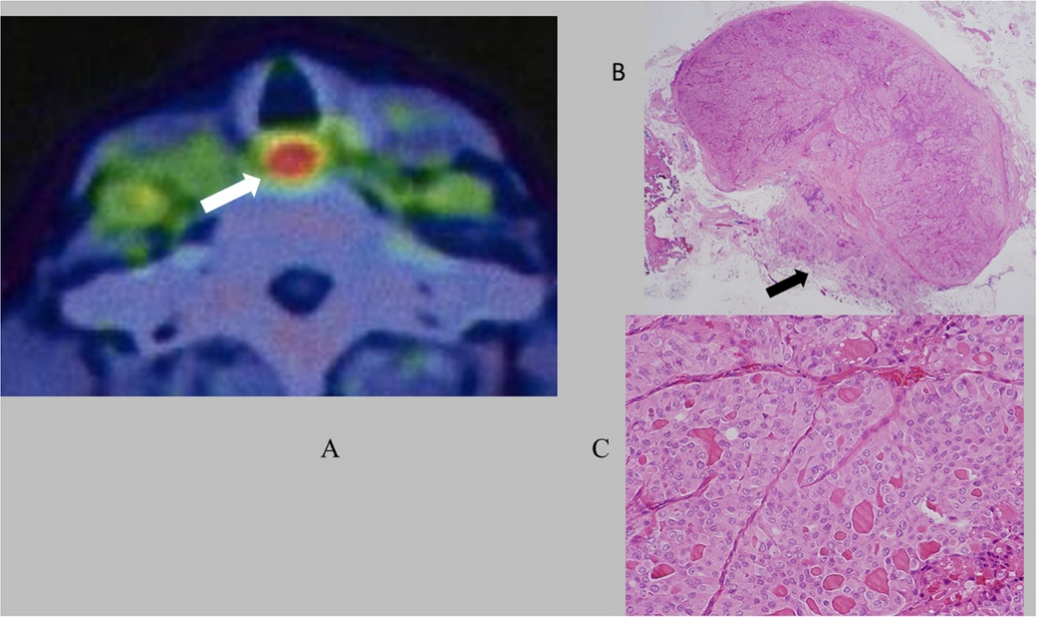
**PET/CT and pathological findings. (A)** Axial view of PET/CT study shows the recurrent tumor mass between the trachea and esophagus with high uptake of FDG at a peak standardized uptake value of 15.45. **(B)** Low-power magnification; arrow shows extracapsular extension. **(C)** High-power magnification. Pathological findings demonstrate poorly differentiated thyroid carcinoma.

**Figure 3:**
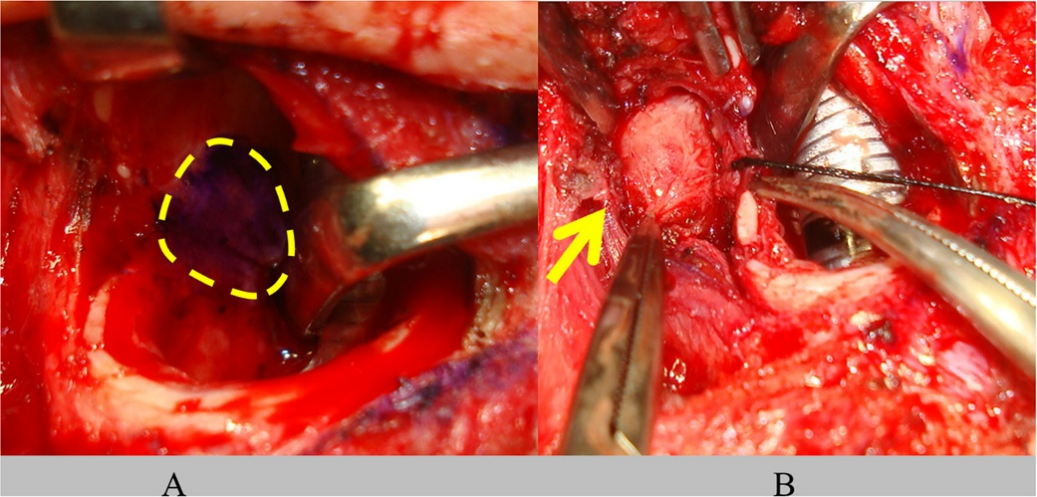
**Intraoperative findings. (A)** The tumor was detected just under the tracheal mucosa. Dashed circle: tumor behind the membranous wall of the trachea. **(B)** The tumor was resected with minimal additional incision of the membranous wall of the trachea. Arrow: pulling out the tumor from between the membranous portion of the retracted trachea and the esophagus.

**Figure 4:**
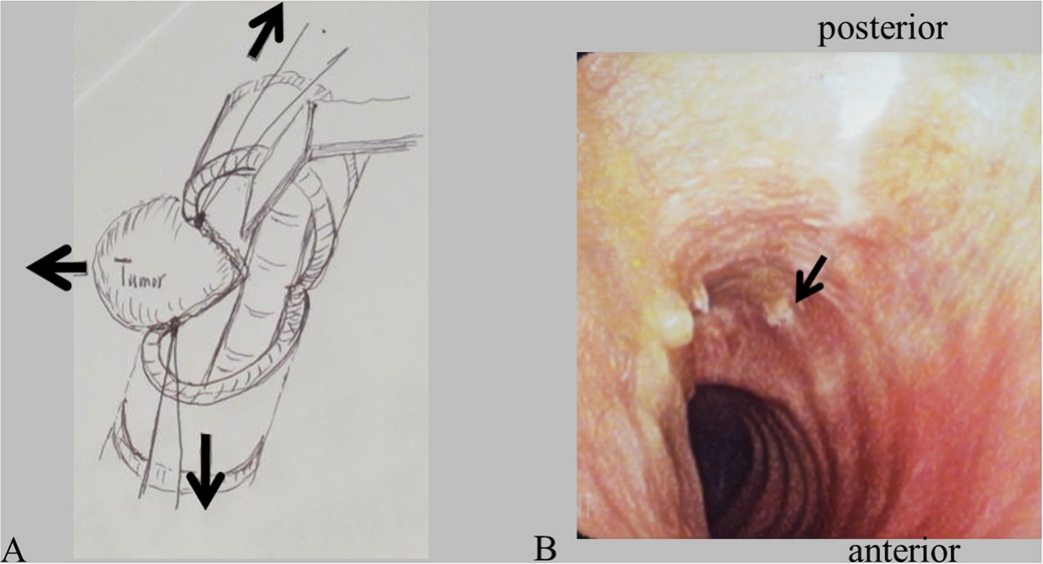
**Surgical procedure and postoperative endotracheal finding. (A)** Surgical procedure. **(B)** Endotracheal finding 3 months after the surgery. There is no granulation formation. Arrow indicates the left-hand end of the surgical scar on the membranous wall of the trachea.

A 69-year-old woman with a past surgical history for a poorly differentiated thyroid cancer presented with a 2 × 2 cm mass between the trachea and the esophagus. The patient already had right recurrent laryngeal palsy from the first treatment. This recurrence was detected by computed tomography (CT), and was shown to be rapidly enlarging over the course of two months (Figure 1). The lesion was also examined by fluorodeoxyglucose (FDG) positron emission tomography (PET), which showed high FDG uptake (maximum standardized uptake value, 15.45) and demonstrated a recurrence of thyroid cancer (Figure 2). After providing written informed consent, the patient underwent surgical resection of the recurrent thyroid cancer with the new procedure. The patient was positioned under general anesthesia in a supine position on the operating table with her neck hyperextended. Through a transverse cervical incision made just along the previous surgical scar, a tracheotomy was performed. The tumor was easily detected through the tracheostoma, then delineated by palpation. The mucous membrane of the trachea was minimally incised along the right-hand border of the tumor and a mucosal flap was elevated (Figure 3). The left-hand side of the trachea including the membranous wall and cartilage of the tracheal mucosa was maximally preserved, to maintain the vascular supply to the trachea. Finally, the membranous wall of the trachea was preserved to within one-third of the left-hand side (Figure 4A). Bleeding from both cut edges of the tracheal mucosa was confirmed. There was no extracapsular spread and the tumor was removed completely with the muscular layer of the esophagus. Intraoperative frozen examination revealed that the tumor was completely resected. A right sternocleidomastoid muscle flap was elevated and inserted into the cavity resulting from the tumor resection and sutured between the esophagus and the trachea. The membranous wall of the tracheal mucosa was also sutured submucosally. A temporary tracheocutaneous stoma was made.

## Results

The tumor was removed completely with the muscular layer of the esophagus without injuring the intact recurrent laryngeal nerve and lateral major vessels. The total surgical time was 170 minutes with 30 ml blood loss. All the removed tissues were sent for histopathologic evaluation, and were found to be consistent with poorly differentiated thyroid carcinoma (Figure 2B). The patient started oral nutritional intake on the first postoperative day, and she was discharged on the seventh postoperative day. The patient did not experience any significant postoperative complications, such as dysphagia. By the 3-month follow-up, the healing of the intratracheal mucosa had been completed and no granulation could be seen (Figure 4B). The tracheostoma was closed under local anesthesia. The patient has been alive with no evidence of recurrence for 5 postoperative years.

## Discussion

Poorly differentiated thyroid carcinoma is a group of carcinomas that exists between well differentiated and anaplastic thyroid carcinoma in terms of both morphological appearance and biological behavior [3, 4]. The World Health Organization classification of endocrine tumors separately categorized poorly differentiated and well differentiated thyroid carcinoma in 2004. In 2006, the Turin proposal for an algorithm for the diagnosis of poorly differentiated carcinomas recommended that it be defined as the presence of a solid, trabecular, or insular growth pattern but with the absence of the conventional nuclear features of papillary carcinoma [5, 6]. The principal treatment for this entity is surgical management. However, bilateral recurrent laryngeal palsy is easily caused by surgical procedures, such as heat resulting from electrocautery or traction used to watch tumors and resect tumors. Bilateral vocal cord paralysis is a serious disease, causing life-threatening respiratory distress or swallowing dysfunction. Moreover, a tracheal anastomotic leak potentially causes painful dyspnea resulting from mediastinal emphysema. Since significant morbidities and poor quality of life may result from surgical procedures for recurrent cancer involving the trachea and esophagus, a minimally invasive procedure is needed. However, it was challenging for us to establish an ideal surgical strategy for recurrent thyroid cancer with unilateral vocal cord paralysis. To reduce morbidities associated with extensive resection and to decrease the potential for bilateral recurrent nerve injury, we adopted an endoluminal approach. The approach through a tracheostoma enables the surgeon to identify the tumor site with ease and without causing recurrent laryngeal nerve injury. There is less risk of bleeding from major lateral vessels, or of injuries to the recurrent laryngeal nerves. The disadvantages of the procedure include the need for a temporary tracheostomy and the possibility of granulation. To avoid tracheal granulation, we sutured the tracheal mucosa submucosally. As a result, there was no granulation of the trachea at all.

The best-known minimally invasive surgical procedure is natural orifice transluminal endoscopic surgery (NOTES), which does not require incision of the skin [7]. Several studies have demonstrated the feasibility of transoral thyroidectomy and parathyroidectomy in animal models [8, 9]. Regarding transtracheal surgery, the feasibility of transtracheal evaluation of the pleural cavity using the NOTES procedure and endoluminal repair of the membranous tracheal disruptions has been reported [10]. However, there has been no report in the literature of transluminal surgery for head and neck lesions. Transtracheal surgery is a modification of the NOTES technique and a feasible option for tumors around the central neck structures. The morbidity from extensive resection for patients with thyroid cancer has led to a poor quality of life. To reduce complications and preserve postoperative function, we recommend transtracheal central neck dissection for recurrent thyroid cancers involving the trachea and esophagus to avoid injuries to the lateral major vessels or recurrent laryngeal nerves.

## Conclusion

The new procedure of transtracheal resection for recurrent thyroid cancer involving the trachea and esophagus was useful and safe.

## Consent

Written informed consent was obtained from the patient for publication of this case report and accompanying images. A copy of the written consent is available for review by the editor-in-chief of this journal.

## Abbreviations

CT: computed tomography
FDG: fluorodeoxyglucose
NOTES: natural orifice transluminal endoscopic surgery
PET: positron emission tomography.
